# Exploring the ceRNA network involving AGAP2-AS1 as a novel biomarker for preeclampsia

**DOI:** 10.1038/s41598-024-79224-2

**Published:** 2024-11-09

**Authors:** Fan Lu, Ni Zeng, Xiang Xiao, Xingxing Wang, Han Gong, Houkang Lei

**Affiliations:** 1 Department of Obstetrics, Affiliated Hospital of GuiZhou Medical University, Guiyang, Guizhou China; 2 Department of Hospital infection and control, Affiliated Hospital of GuiZhou Medical University, Guiyang, Guizhou China

**Keywords:** Preeclampsia, Competing endogenous RNA network, Biomarkers, lncRNA, AGAP2-AS1, Gene regulatory networks, Pre-eclampsia

## Abstract

Preeclampsia (PE) is an important research subject in obstetrics. Nevertheless, the underlying mechanisms of PE remain elusive. PE-related expression datasets (GSE96983, GSE96984 and GSE24129) were downloaded from the Gene Expression Omnibus (GEO) database. Firstly, the differentially expressed messenger RNAs (DE-mRNAs), DE-microRNA (DE-miRNAs) and DE-long non-coding RNA (DE-lncRNAs) between PE and control cohorts were identified, and the ceRNA network was constructed. Then candidate hub genes were obtained through five algorithms by the protein-protein intersection (PPI) network of the mRNAs. Further, five hub genes were identified by receiver operating characteristic (ROC) curve and gene expression profiles: DAXX, EFNB1, NCOR2, RBBP4 and SOCS1. The function of 5 hub genes was analyzed and the interaction between drugs and hub genes was predicted. A total of 5 small molecule drugs were predicted, namely benzbromarone, 9,10-phenanthrenequinone, chembl312032, insulin and aldesleukin. AGAP2-AS1 was mainly located in exosome and cytoplasm. Agap2-as1-related regulatory subnetworks were extracted from ceRNA networks which included 41 mRNAs, 2 miRNAs and 1 lncRNA, including the regulated relationship pairs AGAP2-AS1-hsa-miR-497-5p-SRPRB, and AGAP2-AS1-hsa-miR-195-5p-RPL36. In summary, we constructed a competitive endogenous RNA (ceRNA) network to identify five potential biomarkers (DAXX, EFNB1, NCOR2, SOCS1 and RBBP4) of PE. The in-depth analysis of the AGAP2-AS1 regulatory network will help to uncover more important molecules closely related to PE and provide a scientific Reference.

**Supplementary Information:**

The online version contains supplementary material available at 10.1038/s41598-024-79224-2.

## Introduction

Preeclampsia (PE) is a prevalent acute and severe obstetric disorder, characterized by elevated blood pressure and the presence of excess protein in the urine. It can cause severe multiorgan functional damage and adversely affect the health of both mother and child. If left undetected or untreated, it can result in maternal and neonatal mortality, particularly in severe instances, thus emerging as a significant contributor to global maternal and neonatal death rates^1^. The diagnosis of PE is made by a systolic blood pressure of 140mmHg and/or a diastolic blood pressure of 90mmHg after 20 weeks of gestation, combined with urinary protein quantity and urine protein/creatinine ratio of 0.3 or random urinalysis results. In the absence of proteinuria, the diagnostic criteria for PE can also include the presence of organ or system involvement, such as cardiovascular, respiratory, hepatic, renal, hematological, gastrointestinal, neurological, or placental-fetal abnormalities^2,3^. PE impacts 5-8% of pregnancies, correlating with a higher likelihood of negative pregnancy outcomes like placental detachment, preterm delivery, and restricted fetal growth, leading to stillbirth^4,5^. In recent years, the occurrence rate of PE has been rising annually, but the study of its related pathological mechanism is relatively limited. It is currently believed that factors such as the infiltration capacity of placental trophoblast cells and uterine spiral artery remodeling are closely related to the pathogenesis of PE, but the exact mechanism remains unclear^6,7^. Therefore, it is critical to explore the pathologic mechanisms of PE occurrence in order to find effective and accessible clinical biomarkers for PE screening in the field of obstetrics, and then to develop effective PE treatment and prevention measures in the clinic.

Research indicates that less than 2% of the human genome is made up of genes coding for proteins, with the bulk comprising non-coding genes, such as microRNAs (miRNAs) and long non-coding RNAs (lncRNAs)^8^. The significance of non-coding RNAs in regulating genes has gained increasing recognition in recent years. miRNAs, a category of small, naturally occurring single-stranded noncoding RNAs, are crucial in the progression of diseases. LncRNAs, an RNA molecule with a gene length of more than 200 nucleotide units, lack an open-reading coding frame and have no ability to encode proteins, but they possess a broad array of biological functions, capable of regulating gene expression both at the transcriptional and post-transcriptional stages^9^. LncRNAs play a role in numerous cellular activities, including cell differentiation, immune responses, metastasis in cancer cells, proliferation, and resistance to drugs^10–12^. In addition, lncRNAs can be involved in several biological processes including growth and development, and material metabolism at the epigenetic level^13^. It has been established that lncRNAs and miRNAs are critical in the onset and progression of PE^14–16^. There is a variety of miRNAs expressed differently in the placentas of patients with PE compared to normal pregnancies, suggesting that these miRNAs may be associated with PE^17^. Furthermore, some studies have indicated that lncRNAs can be involved in PE progression by affecting the function of trophoblast cells^18,19^.

Recently, the significance of non-coding RNAs (ncRNAs), including lncRNAs and miRNAs, in gene regulation has increasingly been acknowledged. LncRNAs, a type of RNA molecule exceeding 200 nucleotides in length, don’t encode proteins but are crucial in regulating gene expression^20^. LncRNAs can regulate the transcriptional, post-transcriptional and epigenetic levels of gene expression by interacting with DNA, RNA and proteins^21^. Conversely, miRNAs are small RNA molecules, approximately 22 nucleotides long, primarily regulating gene expression by binding to the mRNAs of target genes, leading to their degradation or inhibiting their translation^22^.

Abnormal expression and dysfunction of lncRNAs and miRNAs have been widely observed during disease development, and they can influence cell proliferation, apoptosis, differentiation, and cell cycle regulation, thus affecting disease onset and progression^23,24^. In the context of PE, numerous research investigations have proposed that lncRNAs and miRNAs might play crucial roles in the onset and advancement of this condition^25,26^. For instance, a previous discovered found that the lncRNA AGAP2-AS1 was aberrantly expressed in tumor cells and was involved in tumorigenesis and progression^27^. In addition, down-regulated lncRNA AGAP2-AS1 causes pre-eclampsia by impairing the trophoblast phenotype as a competing endogenous RNA for jun dimerization protein 2 (JDP2)^28^. However, the specific regulatory mechanism and its specific role manages to be investigated for other downstream of AGAP2-AS1 in PE occurrence.

In this investigation, the transcriptomic data and pertinent clinical data of placental tissues (GSE96983, GSE96984, GSE24129) were acquired from the Gene Expression Omnibus (GEO) repository, specifically focusing on individuals diagnosed with PE. The lncRNA-miRNA-mRNA regulatory network of PE was constructed. Furthermore, PE-related biomarkers were identified by bioinformatics analysis. By deeply studying the function and regulatory mechanism of AGAP2-AS1, our objective is to elucidate the precise involvement of these factors in the onset and progression of PE, thus establishing a significant theoretical foundation for the timely detection and management of PE. In addition, by analyzing the regulatory network of AGAP2-AS1, we anticipate discovering other important molecules related to PE and further resolve the pathogenesis of PE.

## Results

### Creation of ceRNA network

**Fig. 1:**
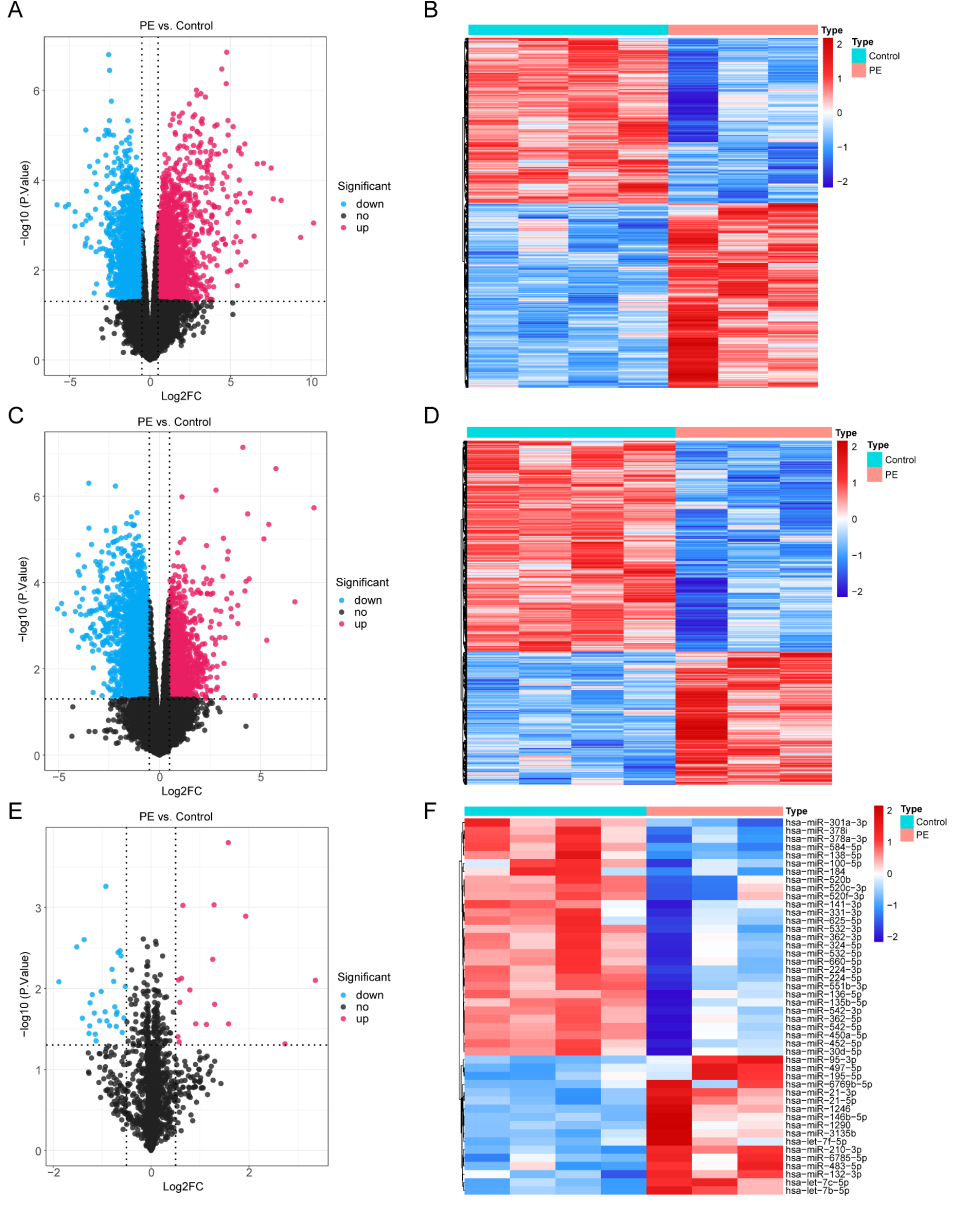
Identification of DE-mRNAs, DE-lncRNAs and DE-miRNAs. The volcano plot (**A**) and heatmap (**B**) of DE-mRNAs between PE and control cohorts. The volcano plot (**C**) and heatmap (**D**) of DE-lncRNAs between PE and control cohorts. The volcano plot (**E**) and heatmap (**F**) of DE-miRNAs between PE and control cohorts.

**Fig. 2:**
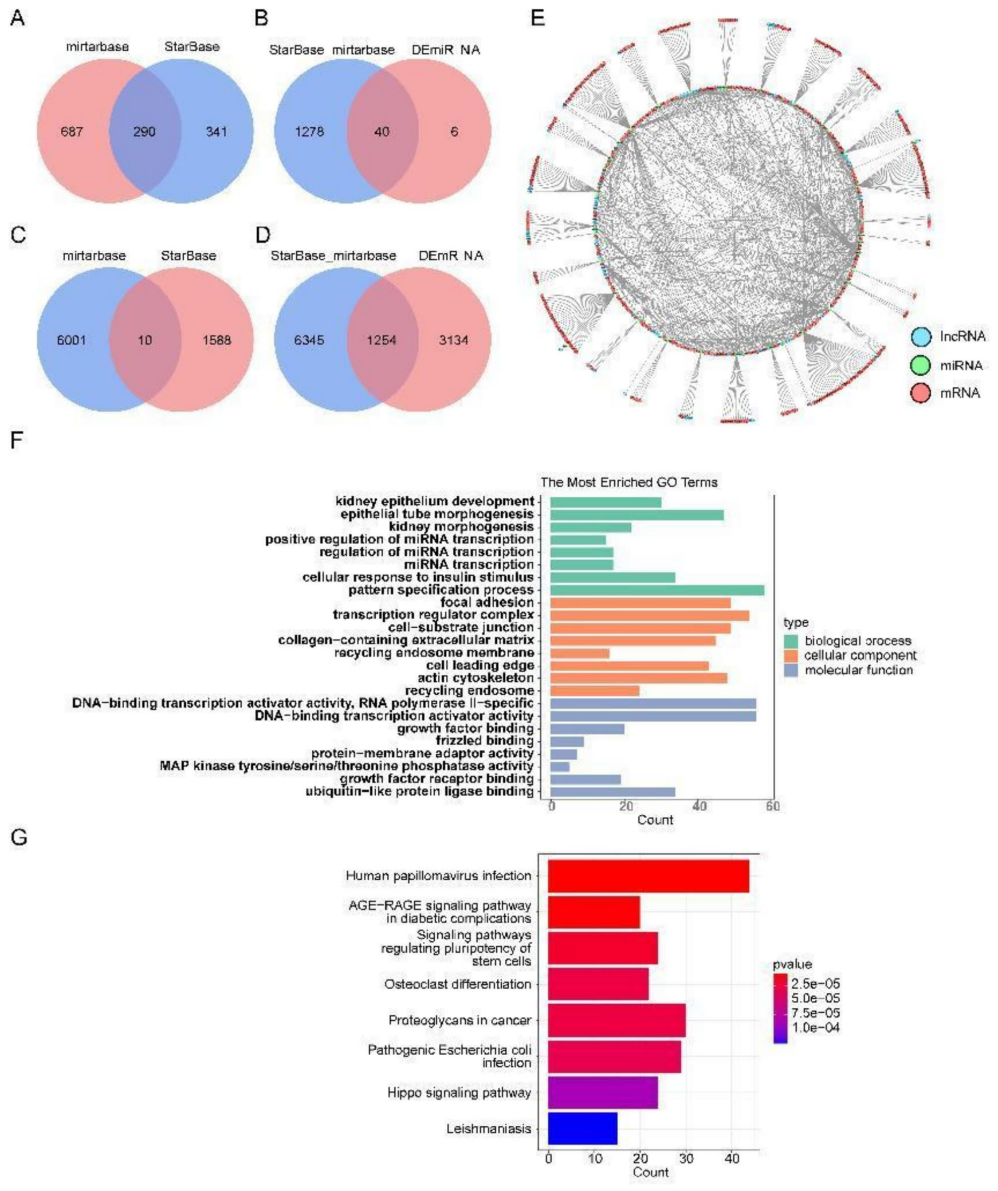
Construction of ceRNA network. (**A**) The Venn map of candidate miRNA between mirtarbase and StarBase database. (**B**) The Venn map between candidate miRNA and DE-miRNA. (**C**) The Venn map of candidate mRNA between mirtarbase and StarBase database. (**D**) The Venn map between candidate mRNA and DE-mRNA. (**E**) Construction of ceRNA network. Blue circles indicate lncRNA, green circles indicate miRNA, red circles indicate mRNA. (**F**) GO enrichment analysis of mRNAs. (**G**) KEGG enrichment analysis of mRNAs.

There were 4388 differentially expressed messenger RNAs (DE-mRNAs) between PE and control cohorts (Fig. 1A,B). In total, 4129 differentially expressed long non-coding RNAs (DE-lncRNAs) (PE vs. control) were identified, comprising 1597 up-regulated lncRNAs and 2532 down-regulated lncRNAs (Fig. 1C,D). There were 46 differentially expressed microRNAs (DE-miRNAs) (PE vs. control), consisting 17 up-regulated miRNAs and 29 down-regulated miRNAs (Fig. 1E,F). There were 1318 miRNAs predicted, and 40 miRNAs were obtained finally (Fig. 2A,B). In total, 7599 mRNAs were predicted, and 1254 mRNAs finally were got (Fig. 2C,D). Afterwards, the ceRNA network was created, including 1203 mRNAs, 39 miRNAs, and 138 lncRNAs (Fig. 2E). For instance, the regulated relationship pairs included AC092168.2-hsa-miR-324-5p-RBBP4, and ADAMTSL4-AS1-hsa-miR-331-3p- SOCS1 (Fig. 2E). The results of enrichment analysis for the mRNAs suggested that, 1517 Gene Ontology- biological process (GO BP) items, 87 Gene Ontology- cellular component (GO CC) items, 113 Gene Ontology-molecular function (GO MF) items, and 85 Kyoto Encyclopedia of Genes and Genomes (KEGG) pathways were enriched (Supplementary Tables 1–2), including kidney epithelium development, focal adhesion, DNA-binding transcription activator activity, human papillomavirus infection, etc. (Fig. 2F,G).

### Acquisition of biomarkers

**Fig. 3:**
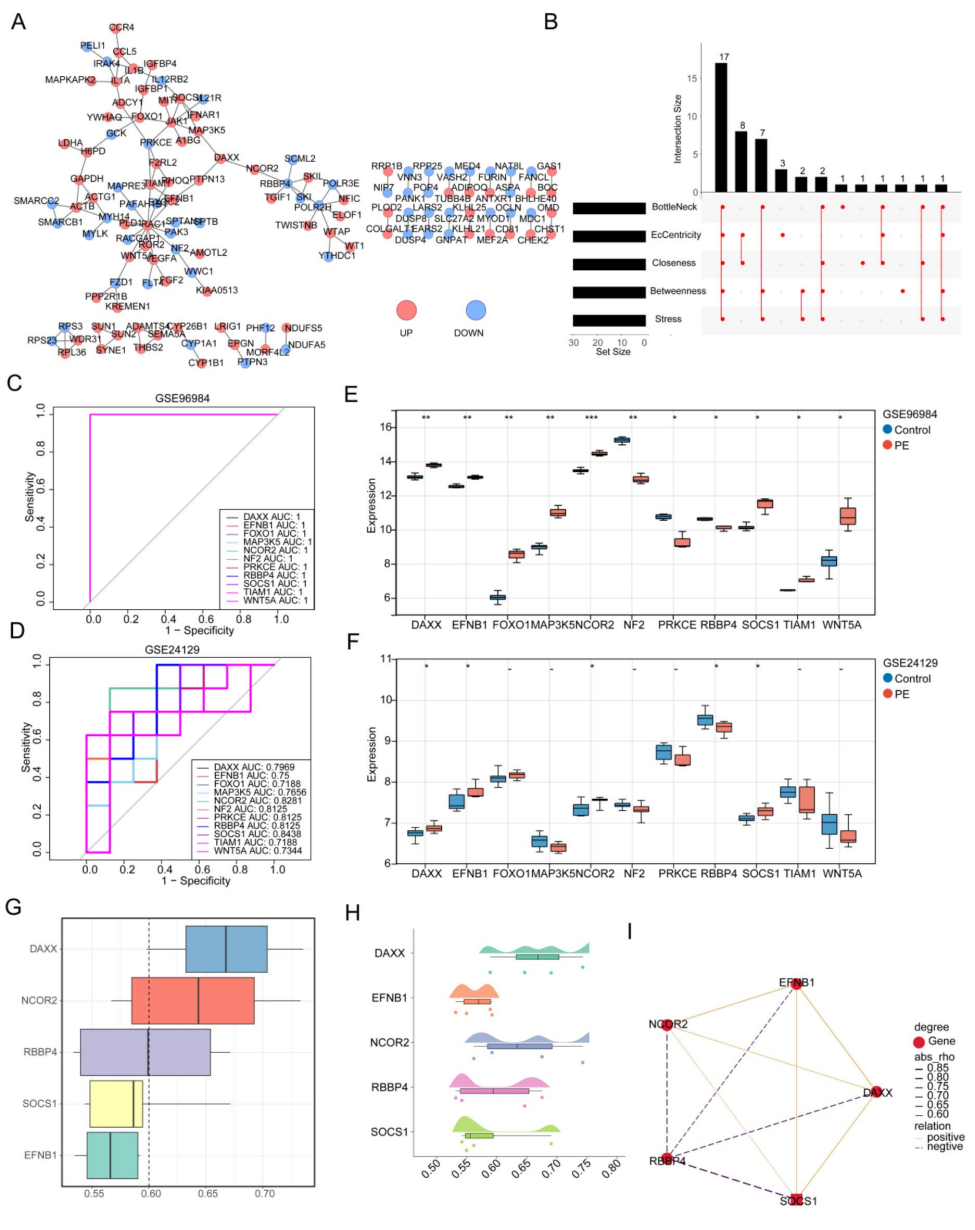
Screening of biomarkers. (**A**) Construction of PPI network. Red circles indicate up-regulated mRNAs, blue circles indicate down-regulated mRNAs. (**B**) The hub gene was obtained by BottleNeck, EcCentricity, Closeness, Betweenness and Stress algorithms. ROC curve of hub genes in GSE96984 (**C**) and GSE24129 dataset (**D**). The expression of hub genes between PE and control cohorts in GSE96984 (**E**) and GSE24129 dataset (**F**). (**G**) GO semantic similarity box plot of hub genes. (**H**) Raincloud plot of relatedness of the GO terms. (**I**) The diagram depicts Pearson correlations between hub genes. ns, not significant; *, *P* < 0.05; **, *P* < 0.01; ***, *P* < 0.001; ****, *P* < 0.0001.

The protein-protein interaction (PPI) network of the mRNAs contained 128 nodes and 123 edges, which displayed that RAC1 interacted with various proteins, including ROR2, PLD1, EFNB1, etc. (Fig. 3A). Then, 17 hub genes were identified, namely RAC1, PRKCE, TIAM1, JAK1, EFNB1, DAXX, PTPN13, MAP3K5, NCOR2, RBBP4, MYH14, ADCY1, SOCS1, FOXO1, WNT5A, NF2, and VEGFA (Fig. 3B). And 11 candidate genes were gained, namely DAXX, EFNB1, FOXO1, MAP3K5, NCOR2, NF2, PRKCE, RBBP4, SOCS1, TIAM1, and WNT5A (Fig. 3C,D). The expression of DAXX, EFNB1, NCOR2, SOCS1, and RBBP4 between PE and control cohorts were significantly different, and showed the same expressive trend in GSE96984 and GSE24129 (Fig. 3E,F). In PE, the expression of DAXX, EFNB1, NCOR2, and SOCS1 was found to be increased, whereas RBBP4 exhibited decreased expression. Therefore, these five genes were defined as the biomarkers. The results of functional similarity demonstrated that the similarity scores of DAXX and NCOR2 were greater than 0.6, indicating these two genes had a strong similarity (Fig. 3G,H). Finally, the relevant analysis showed all five biomarkers correlated with each other, and RBBP4 was negatively correlated with NCOR2, EFNB1, DAXX, and SOCS1 (Fig. 3I). However, NCOR2, EFNB1, DAXX, and SOCS1 were positively correlated with each other (Fig. 3I).

### Enrichment analysis

**Fig. 4:**
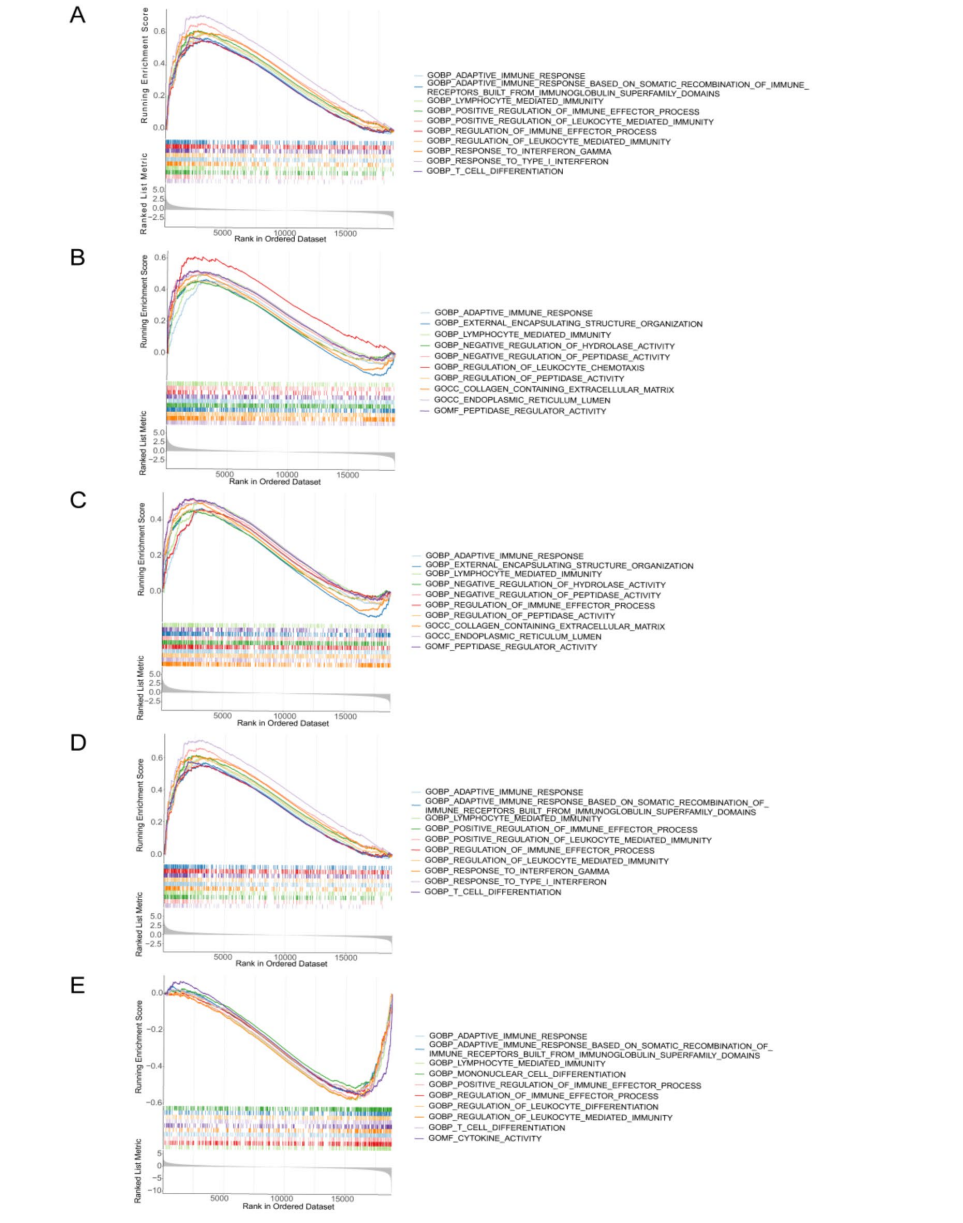
GO enrichment analysis of five hub genes. DAXX (**A**), EFNB1 (**B**), NCOR2 (**C**), SOCS1 (**D**) and RBBP4 (**E**).

**Fig. 5:**
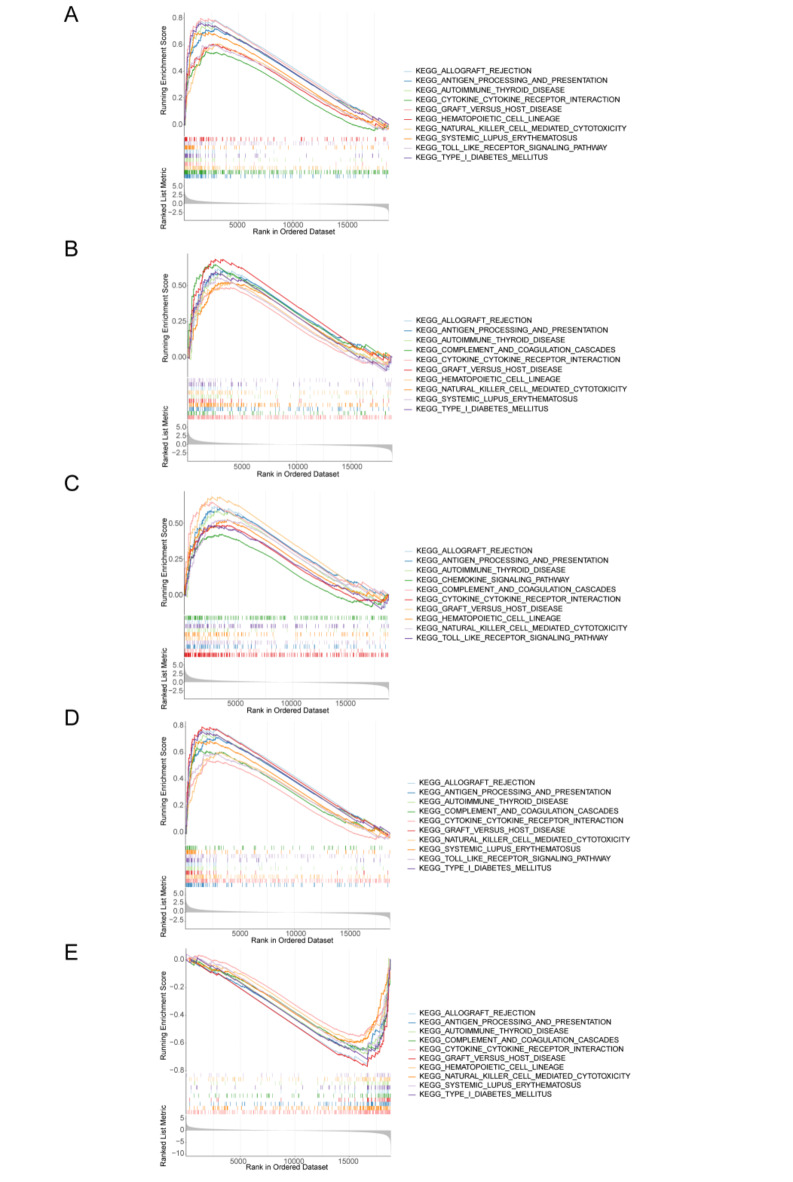
KEGG enrichment analysis of five hub genes. DAXX (**A**), EFNB1 (**B**), NCOR2 (**C**), SOCS1 (**D**) and RBBP4 (**E**).

The enrichment analysis displayed the top 10 GO categories and KEGG pathways. Among the GO results, all biomarkers were involved in lymphocyte mediated immunity and adaptive immune response (Fig. 4A-E). The DAXX, EFNB1, NCOR2, and SOCS1 were involved in these items in high expressed cohort, yet RBBP4 enriched in these items in low expressed cohort (Fig. 4A-E). The DAXX and SOCS1 were involved in positive regulation of immune effector process, T cell differentiation, and regulation of leukocyte mediated immunity in high expressed cohort, while RBBP4 was involved in these items in low expressed cohort (Fig. 4A,D,E). Interestingly, DAXX and SOCS1 enriched in the same items, such as response to interferon gamma, positive regulation of leukocyte mediated immunity, etc. (Fig. 4A,D). Additionally, EFNB1 and NCOR2 also enriched in the same items, such as endoplasmic reticulum lumen, collagen containing extracellular matrix, regulation of peptidase activity, etc. (Fig. 4B,C). Of the KEGG results, all biomarkers were involved in allograft rejection, antigen processing and presentation, graft versus host disease, natural killer cell mediated cytotoxicity, cytokine-cytokine receptor interaction, autoimmune thyroid disease (Fig. 5A,B). The DAXX, EFNB1, NCOR2, and SOCS1 were involved in these items in high expressed cohort, yet RBBP4 enriched in these items in low expressed cohort (Fig. 5A-E). The DAXX, NCOR2, and SOCS1 were involved in Toll like receptor signaling pathway in high expressed cohort (Fig. 5A,C,D). EFNB1 and RBBP4 enriched in the same items, such as type I diabetes mellitus, systemic lupus erythematosus, hematopoietic cell lineage, etc. (Fig. 5B,E). And EFNB1 was involved in these pathways in high expressed cohort, while RBBP4 enriched in these pathways in low expressed cohort (Fig. 5B,E). We speculated that the reason for this result might be due to too few samples in the training set or similar genes functions.

### Drug analysis

**Fig. 6:**
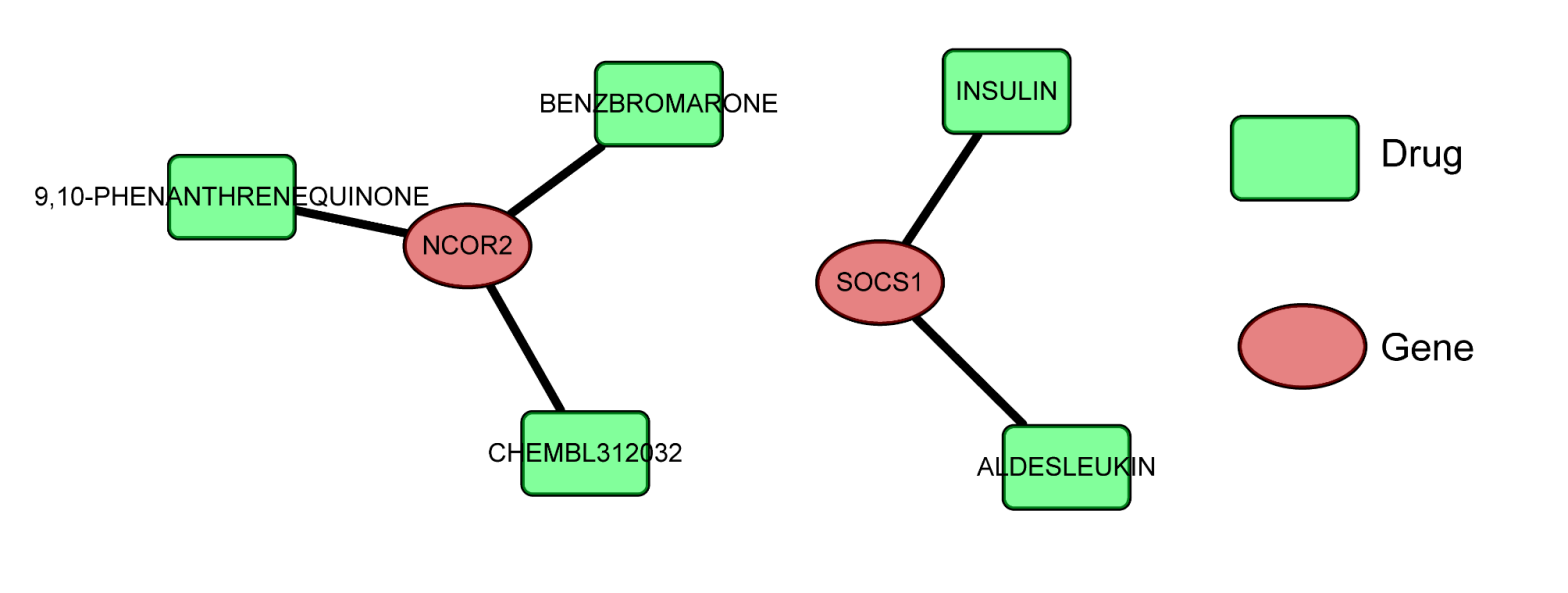
Construction of drug-gene interaction networks. Green squares indicate drug, red circles indicate hub genes.

We generated drug-gene interaction networks using the DGIdb database. The analysis revealed predictions for a total of 5 small molecule drugs (Table 1). There were 3 small molecule drugs predicted for NCOR2, namely benzbromarone, 9,10-phenanthrenequinone, and chembl312032 (Fig. 6). For SOCS1, 2 small molecule drugs were predicted, namely insulin and aldesleukin (Fig. 6). However, no small molecule drugs that were predicted interacted with DAXX, EFNB1, and RBBP4.

**Table 1:** Drug information corresponding to hub gene.

Gene	Drug	Sources	PMID
NCOR2	9,10-PHENANTHRENEQUINONE	DTC	
NCOR2	CHEMBL312032	DTC	
NCOR2	BENZBROMARONE	DTC	
SOCS1	INSULIN	NCI	18,171,911
SOCS1	ALDESLEUKIN	NCI	12,928,391

### Regulatory network and subcellular localization of AGAP2-AS1

**Fig. 7:**
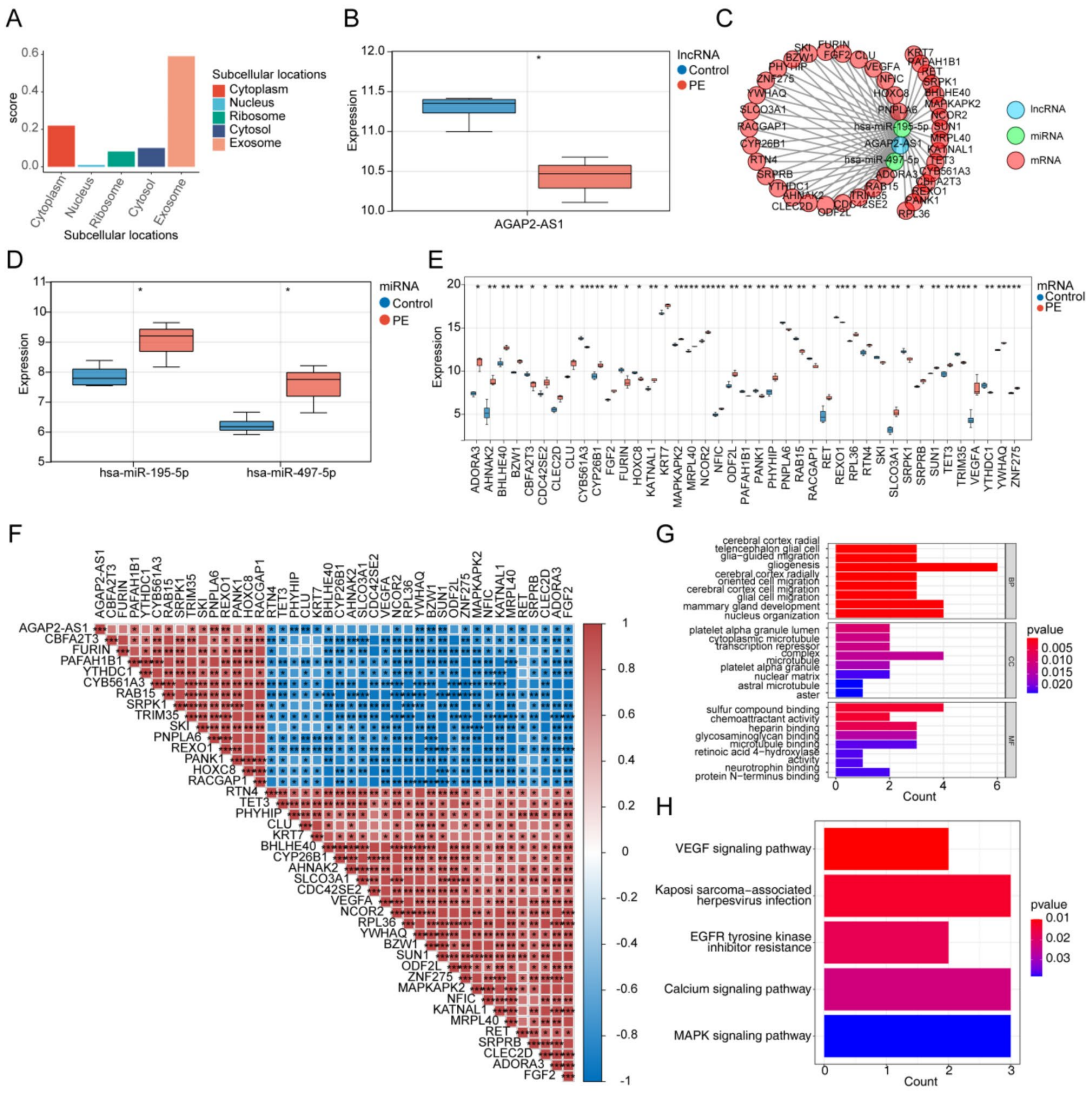
Construction of AGAP2-AS1 regulatory subnetwork. (**A**) Subcellular localization of AGAP2-AS1. (**B**) The expression of AGAP2-AS1 between PE and control cohorts. (**C**) Construction of AGAP2-AS1 regulatory network. Blue circles indicate lncRNA, green circles indicate miRNA, red circles indicate mRNA. (**D**) The expression of hsa-miR-195-5p and hsa-miR-497-5p between PE and control cohorts. (**E**) The expression of mRNA between PE and control cohorts. (**F**) The heatmap of correlation between AGAP2-AS1 and mRNA. (**G**) GO enrichment analysis of mRNAs. (**H**) KEGG enrichment analysis of mRNAs. ns, not significant; *, *P* < 0.05; **, *P* < 0.01; ***, *P* < 0.001; ****, *P* < 0.0001.

The subcellular localization analysis indicated that AGAP2-AS1 predominantly localized within exosomes and the cytoplasm (Fig. 7A). The AGAP2-AS1 expression between PE and control cohorts was significantly different, and markedly lower in the PE cohort (Fig. 7B). Subsequently, we found the constructed ceRNA network containing AGAP2-AS1, and the regulatory network of AGAP2-AS1 was extracted (Fig. 7C). The regulatory network included 41 mRNAs, 2 miRNAs, and 1 lncRNA, which contained the regulated relationship pairs AGAP2-AS1-hsa-miR-497-5p-SRPRB, and AGAP2-AS1-hsa-miR-195-5p-RPL36 (Fig. 7C). Significant differences were observed in the expression levels of both miRNAs and mRNAs between the PE and control cohorts (Fig. 7D-E). The 2 miRNAs were both up-regulated in PE cohorts, and most mRNAs were also significantly up-regulated in PE cohort (Fig. 7D-E). Correlation analysis showed that AGAP2-AS1 had a significant strong or extremely strong correlation with 27 mRNAs, such as PAFAH1B1, SKI, PNPLA6, etc. (Fig. 7F).

The results of enrichment analysis of the mRNAs demonstrated that there were 415 GO BP, 23 GO CC, 28 GO MF, and 5 KEGG pathways enriched (Supplementary Tables 3–4), including cerebral cortex radial glia-guided migration, platelet alpha granule lumen, sulfur compound binding, VEGF signaling pathway, etc. (Fig. 7G-H).

### Validation of the expression of biomarkers in clinical samples

**Fig. 8:**
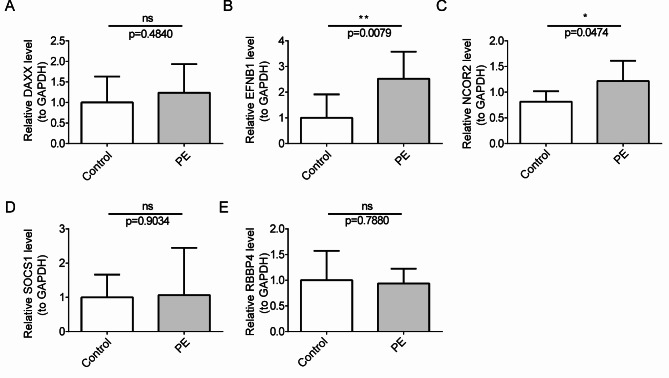
RT-qPCR analysis of expression levels of five hub genes in serum and placenta samples between PE and control cohorts. DAXX (**A**), EFNB1 (**B**), NCOR2 (**C**), SOCS1 (**D**) and RBBP4 (**E**). The error bar indicates standard error (SE). ns, not significant; *, *P* < 0.05; **, *P* < 0.01; ***, *P* < 0.001; ****, *P* < 0.0001.

The expression of biomarkers was further assessed via real time quantitative-Polymerase Chain Reaction (RT-qPCR). DAXX, EFNB1, NCOR2, and SOCS1 were highly expressed and RBBP4 was lowly expressed in the PE cohort (Fig. 8A-E). Nevertheless, the expression levels of DAXX, SOCS1, and RBBP4 did not exhibit substantial variations between the PE and control cohorts (Fig. 8A,D,E). Overall, the expression trends were in line with findings from public database.

## Discussion

PE is a significant pregnancy complication and has emerged as a primary cause of maternal and neonatal deaths worldwide due to its unknown mechanism and poor drug treatment, exploring the potential biomarkers related to its pathogenesis is of important for the prevention and diagnosis of PE^29^. PE is also associated with maternal and neonatal health problems in pregnant women, such as maternal-associated chronic hypertension, myocardial ischemia, end-stage renal disease, placental abruption, intrauterine fetal growth restriction, neonatal-associated bronchopulmonary dysplasia, and cognitive impairments^30^. Moreover, PE is associated with maternal and neonatal health problems in pregnant women, such as maternal-associated chronic hypertension, myocardial ischemia, end-stage renal disease, intrauterine fetal growth restriction, neonatal-associated bronchopulmonary dysplasia and cognitive impairment^31,32^.

In this study, we downloaded PE GeneChip datasets GSE96983, GSE96984 and GSE24129 from the GEO database, and screened 251 DEGs by bioinformatics analysis and validation of the RFalgorithm, followed by downscaling of the differential genes to obtain 17 candidate hub genes, including Ras-related C3 botulinum toxin substrate 1 (RAC1), protein kinase C epsilon (PRKCE), T-lymphoma invasion and metastasis 1 (TIAM1), Janus kinase 1 (JAK1), ephrin B1 (EFNB1), Death domain-associated protein (DAXX), protein tyrosine phosphatase non-receptor type 13 (PTPN13), mitogen-activated protein kinase kinase kinase 5 (MAP3K5), nuclear receptor corepressor 2 (NCOR2), RB binding protein 4 (RBBP4), myosin heavy chain 14 (MYH14), adenylate cyclase 1 (ADCY1), Suppressor of cytokine signaling (SOCS1), Forkhead box O1 (FOXO1), Wnt family member 5 A (WNT5A), Nuclearrespiratoty factor 2 (NF2), and vascular endothelial growth factor A (VEGFA). The 17 candidate keys were analyzed in other datasets GSE96984 and GSE24129 for the expression of candidate hub genes, resulting in 5 hub genes with significant differences and consistent expression pattern, namely DAXX, EFNB1, NCOR2, RBBP4, and SOCS1.

Previous research reveals that the DAXX gene mRNA is a newly identified histone chaperone playing a role in the regulation of chromatin architecture and that DAXX is associated with trophoblast differentiation during placental development, with significantly elevated expression in patients with PE^33,34^. Sabri N et al. demonstrated that tumor suppressor Speckle-type POZ protein regulates tumor progression by modulating the polyubiquitination activity of DAXX^35^. Additionally, treatment with the HPV16 E7 protein can increase the expression of DAXX protein. Conversely, disrupting DAXX expression and using agonists for JNK can diminish the inhibitory effects of HPV16 E7 on TNF-α-induced apoptosis. This indicates that the DAXX/JNK pathway might be implicated in the anti-apoptotic function of HPV16 E7 ^36^. Currently, no literature has been found linking NCOR2 with preeclampsia. We are the first to identify NCOR2 as a biomarker associated with PE, thereby expanding research in this field and providing new therapeutic targets. In our study, the similarity scores of DAXX and NCOR2 genes were greater than 0.6, indicating that these two genes are strongly similar and both are upregulated in PE.

EFNB1 (Ephrin-B1) is expressed in chorionic capillary endothelial cells during early and mid-gestation. Inhibition of EFNB1 suppresses HTR8/SVneo cell invasiveness through downregulation of MMP2 and MMP9; and impairs spiral artery remodeling by decreasing placental growth factor expression, leading to PE pathogenesis^37,38^. Previous studies have indicated that EFBN1 plays a vital role in promoting autophagy in colonic epithelial cells, thereby contributing to the maintenance of intestinal homeostasis and the regulation of blood pressure in humans^39,40^.

There are no reports have detected the association of RBBP4 with the pathogenesis of PE. However, it has been surfaced that RBBP4 is involved in the overlapping functions of the regulation of cell proliferation, apoptosis, and the deposition of histone H3.3 during the preimplantation development of mouse embryos, which is thought to be a crucial component in the pathogenesis of PE^41–43^. Liu H et al. showed that SOCS1 negatively regulates the JAK/STAT1 signaling pathway and affects placental trophoblast invasiveness, and the assessment of clinical specimens revealed elevated levels of SOCS1 and IFN-γ expression in patients diagnosed with PE compared to those in the healthy control cohort^44^. Mayor-Lynn K et al. showed that SOCS1 can alter normal placental function by regulating cellular activity, leading to the development and progression of PE^45^. Our study analysis demonstrated a significant upregulation of EFNB1 and SOCS1, as well as a notable downregulation of RBBP4, in PE, corroborating the aforementioned observations.

The DGIdb database predicts the interaction of drugs with the hub gene and finds 5 potential small molecule drugs for the treatment of eclampsia, which provides new ideas for the treatment of eclampsia. We identified a total of five target drugs 9,10-PHENANTHRENEQUINONE, HEMBL312032, BENZBROMARONE, INSULIN, ALDESLEUKIN, which could be influential in the future management of individuals suffering from PE. INSULIN has been reported to be involved in the pathogenesis of PE, and its distinct process might be linked to the crucial function of insulin signaling in the differentiation, longevity, and effector actions of immune cells, primarily through the standard activation of the PI3K/Akt/mTOR pathway^46,47^. Hyperinsulinemia, characterized by insulin resistance or aggressive insulin treatment, might directly contribute to immune cell impairment, which is a key factor in the onset of PE^48,49^.

We extracted the AGAP2-AS1-related regulatory sub-network from ceRNA network and performed Gene Set Enrichment Analysis (GSEA) analysis. AGAP2-AS1, a 1567 nucleotide lncRNA situated on chromosome 12q14.1, has been identified as being unusually expressed in multiple diseases, such as cancer^50,51^. Decreased expression of AGAP2-AS1 in PE has been previously reported, whereas overexpression of AGAP2-AS1 in cells enhances the growth, invasion, and movement of trophoblast cells and hinders the programmed cell death of these cells.AGAP2-AS1 stabilizes trophoblast cells and reduces apoptosis by sponging the miR-574-5p Promoter-Depressor Protein Jun Dimerization Protein 2 (JDP2), and our findings are consistent with previous reports^28^. In brain tumor research, AGAP2-AS1 is involved in the regulation of the Wnt signaling pathway through its interaction with miR-15a/b-5p, thereby influencing the pathogenesis, diagnosis, prognosis, and treatment of brain tumors^52^. Another study on renal cell carcinoma (RCC) revealed a different pathway, where IGF2BP3 stabilizes AGAP2-AS1, allowing it to competitively bind with miR-9-5p, consequently upregulating the expression of Thrombospondin-2 (THBS2) and activating the PI3K/AKT signaling pathway. This process induces M2 polarization of macrophages and promotes the progression of RCC^53^. Additionally, in studies on glioblastoma (GBM), AGAP2-AS1 in exosomes (Exo-AGAP2-AS1) was found to inhibit the function of miR-486-3p through a sponging mechanism, thereby regulating the expression of Transforming Growth Factor β1 (TGF-β1) in myeloid-derived suppressor cells (MDSCs). This regulatory mechanism promotes the development of GBM cells and provides new potential targets for GBM treatment^54^. We followed up with GSEA analysis, and we found that AGAP2-AS1 was mainly enriched in the VEGF pathway as well as the EGFR pathway and MAPK pathway. Previously, it has been reported in the literature that VEGF is involved in the formation of gestational diabetes and peeclampsia^55,56^. MAPK has been used as an important pathway for stabilizing trophoblast cells and an important mechanism in the pathogenesis of PE^57^. Our study is consistent with previous reports. By correlation analysis, we found that AGAP2-AS1 may affect the pathogenesis of PE by regulating genes such as TRIM35 to influence the expression of DAXX, EFNB1, NCOR2, RBBP4 and SOCS1.

Research has found that lncRNAs in biological fluids, as promising biomarkers, have shown significant emerging relevance in the risk stratification of pregnancy-related complications (PRC), including preeclampsia (PE). Studies on the expression patterns of lncRNAs and their potential clinical applications hold diagnostic, prognostic, and therapeutic value, paving the way for innovative approaches to improve prenatal care and the prognosis of pregnant women and fetuses^58^. Additionally, Xiao-Hong Wei and others have discovered that the long non-coding RNA (lncRNA) DUXAP8 is upregulated in the placental tissues of patients with preeclampsia and is significantly correlated with multiple clinical indices. This finding reveals the crucial role of DUXAP8 in regulating the biological behavior of trophoblasts through the FAM134B-mediated endoplasmic reticulum (ER) phagocytosis process, thus providing new theoretical support and perspectives for exploring the pathogenesis of preeclampsia (PE)^59^.However, further studies are needed to determine how LNC RNA affects the development of PE. Currently, although there is no direct evidence to suggest an interaction between lncRNA AGAP2-AS1 and the five core genes, existing research has revealed that the downregulation of lncRNA AGAP2-AS1 could act as a key inhibitory factor in preeclampsia (PE) by competitively inhibiting JDP2 at the post-transcriptional level through miR-574^28^. Given the direct or indirect association between these five core genes and preeclampsia, it is reasonable to speculate that there might be some as yet undefined but significant connections between AGAP2-AS1 and these core genes. This potential mechanism warrants further investigation, and our current findings lay a theoretical foundation and direct the path for future scientific exploration.

## Conclusion

In this study, we established a ceRNA regulatory network and identified hub genes of PE, namely DAXX, EFNB1, NCOR2, RBBP4 and SOCS1, which provided new ideas for the study of the pathogenesis of PE. However, this study is limited by its small sample size. This may weaken the statistical power and introduce sample bias, thereby introducing uncertainties or errors in the research results, which reduces the credibility of the conclusions. To overcome this limitation, we plan to enhance the statistical power in future research by increasing the sample size. This will help to reduce potential biases and errors, ensuring the scientific integrity and rigor of the experimental design, thereby improving the reliability and scientific validity of the research findings.We will follow up with an in-depth study on how LncRNA regulates the relationship between AGAP2-AS1 and DAXX, EFNB1, NCOR2, RBBP4 and SOCS1.

## Materials and methods

### Data sources

The datasets related to PE (GSE96983, GSE96984, and GSE24129) were retrieved from the GEO repository, accessible through the https://www.ncbi.nlm.nih.gov/. The samples of all three data sets are placental samples. The dataset GSE96983 consists of three samples from individuals with PE and four samples from healthy controls. The dataset GSE96984 comprises three samples from PE subjects and four samples from control subjects. The GSE96983 and GSE96984 datasets were from the same cohort of patients, with patients aged 26, 28, 29, 30, 31, 32, and 33, respectively. The mRNA sequencing data (mRNA-seq) and lncRNA sequencing data (lncRNA-seq) were downloaded from the GSE96984. The miRNA sequencing data (miRNA-seq) was downloaded from the GSE96983. The mRNA-seq was downloaded from the GSE24129 to perform validation, and the GSE24129 included 8 PE samples and 8 control samples^60^. In the GSE24129 dataset, all placenta biopsy samples originated from collections following caesarean section procedures. To ensure that the delivery process did not potentially affect the gene expression profiles of the tissue samples, placentas from women who had never experienced a delivery were purposely selected as the source of the samples.

### Creation of the competing endogenous RNA (ceRNA) network

Firstly, the gene expression matrix of three PE samples and four control samples in the GSE96984 dataset was analyzed by the voom method in the “limma” R package (version 3.48.3) (|log2FC| > 0.5 and *P* < 0.05)^61^, and DE-mRNAs and DE-lncRNAs between the PE groups and the control groups were identified. Subsequently, DE-miRNAs between PE and control were analyzed in the GSE96983 dataset (|log2FC| > 0.5 and *P* < 0.05). Finally, volcano plots were drawn using the “ggplot2” R package (version 3.3.5)^62^ to show DE-lncRNAs, DE-miRNAs and DE-mRNAs, and heatmaps of differential expression were drawn using the “pheatmap” R package (version 1.0.12)^63^. The target miRNAs of DE-lncRNAs were predicted by StarBase (http://starbase.sysu.edu.cn) and miRTarBase database (http://mirtarbase.mbc.nctu.edu.tw), respectively, and the screened miRNAs were taken to intersect to obtain candidate miRNAs, and its intersection with DE-miRNAs was taken to obtain overlapping miRNAs. Similarly, the target mRNAs of DEmiRNAs were predicted by StarBase (http://starbase.sysu.edu.cn) and miRTarBase database (http://mirtarbase.mbc.nctu.edu.tw), respectively, and the screened mRNAs were intersected to obtain candidate mRNAs, whose intersection with DE-mRNAs was taken to obtain overlapping mRNAs. Subsequently, the Cytoscape software (version 3.9.1) was used to integrate the interactions of DElncRNAs and overlapping miRNAs and overlapping mRNAs, to construct the lncRNAs-miRNAs-mRNAs regulatory network with overlapping miRNAs regulating both DElncRNAs and overlapping mRNAs as a complete ceRNA. Finally, the overlapping mRNAs were enriched (*P* < 0.05 and counts ≥ 1) by the “ClusterProfiler” R package (version 4.0.2)^64^ to look for common functions and related pathways. The top 10 GO-enriched functions and KEGG-enriched pathways were plotted using the “ggplot2” R package (version 3.3.5)^65–67^.

### Screening of biomarkers

The STRING database was utilized to construct the PPInetwork of the identified mRNAs, considering only interactions with a confidence score higher than 0.9. Then, the BottleNeck, EcCentricity, Closeness, Betweenness, and Stress in Cytoscape were utilized to identify hub genes. The hub genes were identified by intersecting the top 30 genes obtained from five different algorithms. The hub genes underwent additional screening based on Receiver Operating Characteristic (ROC) curves. Candidate genes were identified among the hub genes based on an area under the curve (AUC) value exceeding 0.7. Subsequently, the expression levels of candidate genes in both PE and control groups were examined using datasets GSE96984 and GSE24129. The candidate genes that were significantly different between PE and control cohorts and had the same expressive trend in both datasets were used for subsequent analysis and noted as biomarkers. The functional similarity among the biomarkers was assessed via “GOSemSim” R package (version 2.18.1)^68,69^. Lastly, the correlation between the identified biomarkers was determined using the Spearman correlation coefficient.

### Functional analysis

To investigate the enrichment pathways associated with the biomarkers, we conducted GSEA using the “clusterProfiler” R package (version 4.0.2) along with org.Hs.eg.db (version 3.13.0)^64^. The reference gene sets used for analysis were obtained by downloading the “C2.cp.kegg.v7.5.1.symbols.gmt” and “C2.go.v7.5.entrez.gmt” datasets. Based on the median expression value of the identified biomarkers, the samples from GSE96984 dataset were categorized into cohorts with high and low expression levels, and differential analysis was carried out. The all genes were ranked via log fold change (logFC). The threshold value were |normalized enrichment score (NES)| > 1, NOM *P* < 0.05, and q < 0.25.

### Prediction of small molecule drugs

Each biomarker was utilized as a keyword to predict potential small molecule drugs that interact with the biomarkers in the Drug-Gene Interaction Database (DGIdb, available at https://dgidb.org). Subsequently, the biomarker-drug network was visualized using Cytoscape software (version 3.7.2)^70^.

### Creation of regulatory network and subcellular localization of AGAP2-AS1

Firstly, the subcellular localization of AGAP2-AS1 was performed through lncLocator Database (www.csbio.sjtu.edu.cn/bioinf/lncLocator). Then, the AGAP2-AS1 expression between PE and control cohorts was compared via Wilcox test. The regulatory network of AGAP2-AS1 was extracted from the constructed ceRNA network. The expression of AGAP2-AS1-regulated miRNAs and mRNAs between PE and control were analyzed. Additionally, the correlation of AGAP2-AS1 with AGAP2-AS1-regulated mRNAs was calculated by Pearson. The 0.0 < |r| < 0.2 was extremely weak or no correlation, 0.2 ≤ |r| <0.4 was weak correlation, 0.4 ≤ |r| < 0.6 was moderate correlation, 0.6 ≤ |r| < 0.8 was strong correlation, and 0.8 ≤ |r| < 1.0 was extremely strong correlation. Afterwards, the enrichment analysis were carried out for mRNAs via “ClusterProfiler” R package (version 4.0.2) (*P* < 0.05 and count ≥ 1)^64^.

### Differential gene correlation analysis with LncRNA regulated genes

In order to delve deeper into the association between lncRNA regulated genes and DE-mRNA in PE, we performed a correlation analysis.Genes with significant positive correlation between LncRNA and DE-mRNA were selected for correlation analysis. Spearman’s correlation analysis was employed to assess the correlation between the expression of DE-mRNA and the data obtained from GEO datasets on the expression of regulatory lncRNAs.

### Sample collection and RT-qPCR

A total of 8 PE patients and 8 controls at Affiliated Hospital of Guizhou Medical University from January 2023 to June 2023 were enrolled in this study.All PE patients were diagnosed according to the 2020 guidelines for hypertensive disorders of pregnancy issued by the American College of Obstetricians and Gynecologists (ACOG)^2^. This study did not involve human in vivo experiments or human transplantation-related studies and has been conducted in accordance with the Declaration of Helsinki, which has been obtained from all participants and informed consent.The exclusion criteria were as follows: pregnant women under the age of 18, smoking, alcohol addiction, history of diabetes, anemia, dyslipidemia, autoimmune diseases, malignancy, or gastrointestinal surgery. After the termination of pregnancy, fetal side placental tissues were immediately collected under sterile conditions, approximately 10*10 mm in size, washed with saline, and rapidly frozen at -80 °C. And the Serum and placenta samples were collected to perform RT-qPCR. This study was approved by Ethics Committee of Affiliated Hospital of Guizhou Medical University, Approval Number 2022(410). All participants provided their informed consent by signing a consent form prior to their involvement in the study. The RNA was extracted by TRIzol (Ambion, Austin, USA) in accordance with the instructions provided by the manufacturer. SureScript-First-strand-cDNA-synthesis-kit (Servicebio, Wuhan, China) was utilized to perform reverse transcription analysis. The RT-qPCR analysis was performed using the 2xUniversal Blue SYBR Green qPCR Master Mix (Servicebio, Wuhan, China). The primer sequences used for the analysis can be found in Table 2. The expression levels of biomarkers were quantified using the 2^−ΔΔCt^ method, with normalization to the mRNA levels of GAPDH^71^.

**Table 2:** The information of primer sequence in RT-qPCR.

Gene	Primer sequence (5’-3’)
DAXX-F	GAAATCCCCACCACTTCCTCC
DAXX-R	GCACGATGATGCTGTTAGCG
EFNB1-F	AGCAGTGGGAGGTTTGTGAG
EFNB1-R	TAGAAGAGCGGGGAGATGCT
NCOR2-F	GATGGTGGGCTCCAAGACTG
NCOR2-R	CCTCATTCCCAGAGGCATGTA
RBBP4-F	CAGCATTCATCGACTTGTCCT
RBBP4-R	TGTGACGCATCAAACTGAGCA
SOCS1-F	GACACGCACTTCCGCACATT
SOCS1-R	CGAGGCCATCTTCACGCTAA
Reference gene-GAPDH-F	CGAAGGTGGAGTCAACGGATTT
Reference gene-GAPDH-R	ATGGGTGGAATCATATTGGAAC

### Data statistics

Statistical analysis was conducted using R software (version 4.2.0) (https://www.r-project.org) and GraphPad Prism v9.0. Differences between two cohorts were analyzed using ANOVA and Wilcoxon’s test for multiple cohorts. The difference in OS between cohorts was estimated using a log-rank test and Kaplan-Meier (K-M) analysis. Pearson’s correlation test was utilized to determine the associations among subtypes, clinicopathological features, risk scores, immune checkpoint expression, methyltransferases, and levels of immune infiltration. The obtained results were deemed statistically significant at a significance level of *P* < 0.05.

## Electronic supplementary material

Below is the link to the electronic supplementary material.

Supplementary Material 1

Supplementary Material 2

Supplementary Material 3

Supplementary Material 4

## Data Availability

The datasets used and analyzed in the current study are available from the GEO database (https://www.ncbi.nlm.nih.gov/gds) [GSE96983, GSE96984 and GSE24129], StarBase (Http://starbase.sysu.edu.cn), miRTarBase Database (Http://mirtarbase.mbc.nctu.edu.tw), DGIdb database (https://dgidb.org), lncLocator Database (www.csbio.sjtu.edu.cn/bioinf/lncLocator).

## Abbreviations

PE: Preeclampsia
ceRNA: Competing endogenous RNA
GEO: Gene Expression Omnibus
DE-mRNAs: Differentially expressed message RNAs
DE-lncRNAs: Differentially expressed long non-coding RNAs
DE-miRNAs: Differentially expressed microRNAs
PPI: Protein-protein intersection
ROC: Receiver operating characteristic
AUC: Area under the curve
lncRNAs: Non-coding RNAs
mRNA-seq: mRNA sequencing
lncRNA-seq: lncRNA sequencing
miRNA-seq: miRNA sequencing
GSEA: Gene Set Enrichment Analysis
NES: Normalized enrichment score
GO: Gene Ontology
CC: Cellular component
BP: Biological process
MF: Molecular function
KEGG: Kyoto Encyclopedia of Genes and Genomes
RT-qPCR: Real time quantitative-polymerase chain reaction
